# Immunogenicity and Immune Silence in Human Cancer

**DOI:** 10.3389/fimmu.2020.00069

**Published:** 2020-03-06

**Authors:** Mark Yarmarkovich, Alvin Farrel, Artemio Sison, Moreno di Marco, Pichai Raman, Joshua L. Parris, Dimitrios Monos, Hongzhe Lee, Stefan Stevanovic, John M. Maris

**Affiliations:** ^1^Division of Oncology and Center for Childhood Cancer Research, Children's Hospital of Philadelphia, Philadelphia, PA, United States; ^2^Perelman School of Medicine at the University of Pennsylvania, Philadelphia, PA, United States; ^3^Department of Biomedical and Health Informatics, Children's Hospital of Philadelphia, Philadelphia, PA, United States; ^4^Department of Immunology, University of Tübingen, Tübingen, Germany; ^5^The Center for Data Driven Discovery in Biomedicine, Children's Hospital of Philadelphia, Philadelphia, PA, United States; ^6^Department of Pathology and Lab Medicine, Children's Hospital of Philadelphia, Philadelphia, PA, United States

**Keywords:** immunoediting, immune evasion, neoantigens, HLA, cancer susceptibility, KRAS, BRAF, TP53

## Abstract

Despite recent advances in cancer immunotherapy, the process of immunoediting early in tumorigenesis remains obscure. Here, we employ a mathematical model that utilizes the Cancer Genome Atlas (TCGA) data to elucidate the contribution of individual mutations and HLA alleles to the immunoediting process. We find that common cancer mutations including BRAF-V600E and KRAS-G12D are predicted to bind none of the common HLA alleles, and are thus “immunogenically silent” in the human population. We identify regions of proteins that are not presented by HLA at a population scale, coinciding with frequently mutated hotspots in cancer, and other protein regions broadly presented across the population in which few mutations occur. We also find that 9/29 common HLA alleles contribute disproportionately to the immunoediting of early oncogenic mutations. These data provide insights into immune evasion of common driver mutations and a molecular basis for the association of particular HLA genotypes with cancer susceptibility.

## Introduction

The immune system is thought to play a dual role in carcinogenesis (1–3). First, when a proper immune response is mounted, the immune system is capable of eliminating neoplastic cells arising from early tumor-initiating events (immunoediting). In contrast, the immune system can initiate signaling of wound healing pathways that can help foster an environment conducive to tumorigenesis. The human leukocyte antigen (HLA) proteins present a snapshot of all nucleated cell's proteomes on the cell surface for surveillance by T cells. While an individual harbors six distinct HLA Class I alleles (A, B, and C), a total of 13,145 unique Class I alleles have been characterized to date at these highly polymorphic loci (4). Presentation of processed pathogen-derived peptide by at least one of these HLA alleles is a prerequisite for the initiation of an adaptive immune response. Each HLA allele possesses the ability to present a distinct set of peptides to the immune system, based on the biophysical properties within the peptide binding groove which restrict specificity to a limited set of available peptides. Peptide binding is largely dictated by two HLA-facing anchor residues, which are restricted to a few amino acids at these positions (5). Recently, algorithms such as NetMHC have allowed the prediction of binding affinity of peptide sequences to specific HLA alleles, resulting in the correct prediction of >75% of binders, with positive predictive values in the range of 90–95% (6–10).

Presented neoantigens can be divided into two distinct classes: group 1 resulting from mutations in the TCR-facing residues and correspondingly less likely to change the binding affinity of the peptide/HLA complex, and group 2 resulting from the anchor residues of the peptide, and thus presenting a longer sequence of novel polypeptides to the immune system, as compared to single-residue alterations in group one antigens (5). A properly mediated interaction between the HLA protein, presented peptide, and T cells serves to maintain the genomic integrity of the organism by eliminating cells harboring foreign genetic material both from external pathogens and those arising from somatic mutations. The tumor immunoediting theory predicts that early pathogenic events giving rise to precancerous cell growths can be eliminated by the adaptive immune system unless cancer cells evolve the ability to escape this selective pressure (11).

While it has become increasingly appreciated that the adaptive immune system has the potential to play a significant role in the elimination of existing tumors, its role in the clearance of cancer cells during early initiating events has remained difficult to study. Though it has been well demonstrated that immunosuppression in humans is linked to an increased incidence of cancer (12–15), it has remained difficult to quantify early immunoediting events, and to attribute the clearance of precancerous lesions in immunocompetent individuals to clearance of tumor-derived neoantigens, as opposed to other mechanisms such as the elimination of cells harboring cancer-inducing viruses.

Here, using the large cohort of patients characterized by the TCGA, we created an immunogenicity map of neoantigens resulting from 125 consensus cancer-related genes then employ a mathematical model to estimate early immunoediting events by quantifying the underrepresentation of specific neoantigens and their potential HLA pairs as a metric for prior clearance of these neoantigens by the immune system. We also employ an HLA presentation score to characterize population-scale HLA presentation along the span of individual proteins to uncover both “protected” (peptides derived from protein regions highly represented across the population by HLA) and “unprotected” regions (those not presented by common HLAs) within proteins commonly mutated in cancer. Further, we introduce an HLA-centric metric of immunoediting, allowing the modeling of the degree of immunoediting in early tumor initiating events that generate strongly immunogenic antigens presented by MHC Class I at a population scale occurring across various point mutations, histotypes, patients, and HLA alleles. We have released a companion web application that can be used to explore immunogenicity of tumor neoantigens across the population and HLA presentation along the span of individual proteins for the identification of shared tumor neoantigens and tumor vaccine design (http://reslnmaris01.research.chop.edu:3838/shinyNAP/).

## Results

### HLA Immunogenicity Map Shows That Common Mutations Generate Peptides That Are Immunogenically Silent

**Figure 1:**
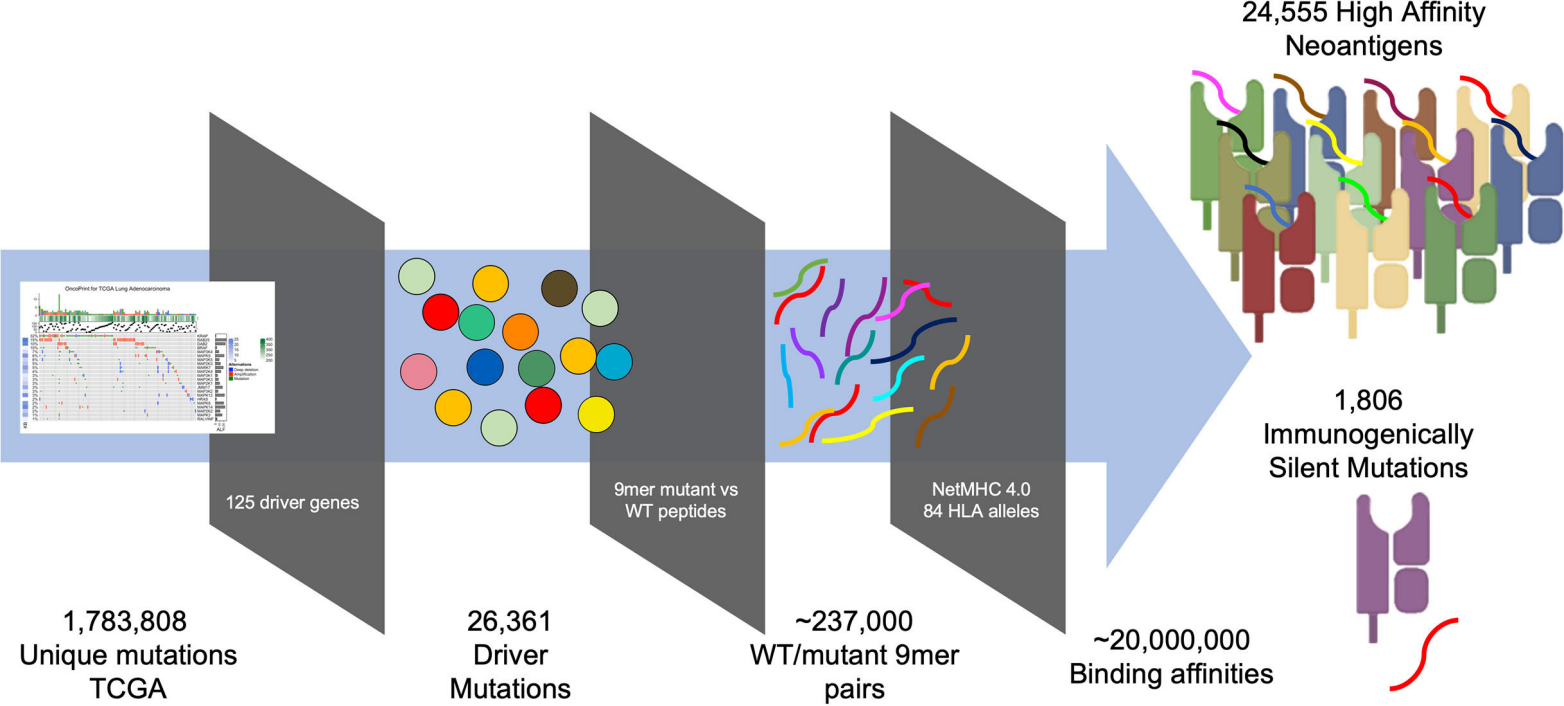
Pipeline for generating immunogenicity map. TCGA mutations imputed by MuSE and Somatic Sniper were filtered for those arising from 125 characterized driver mutations (16). 17 mer peptides sequences were generated for all possible 26,361 variants arising from (covering nine potential epitopes for each variant) each variant/WT pair (~237,000 pairs) and analyzed for MHC binding affinity using NetMHC 4.0 across 84 HLA alleles resulting in 20e^6^ binding affinities. Peptide affinities were aggregated for all peptides arising from each variant and filtered for those that are strong binders (<0.5% rank), revealing 24,555 strong binders and 1,806 immunogenically silent mutations.

To generate a map of immunogenicity across all frequently observed single nucleotide mutations in cancer driver genes, we filtered DNA sequencing data from the TCGA (7,300 subjects representing 33 cancer histologies) for all observed mutations harbored in 125 consensus oncogenes and tumor suppressor genes [(16), Table S1], resulting in 26,361 unique variants (Figure S1). For each variant we then generated 17 mer amino acid sequences to cover all possible 9 mer peptides resulting from amino acid sequences flanking the mutated site (9 potential variants per mutation), and calculated binding affinity using NetMHC-4.0 across 84 common HLA Class I alleles (8), with the alleles studied estimated to represent at least one allele in 99.4% of the US population (calculated based on the ethnicity-adjusted allele frequency from the Bone Marrow Registry) (17). This analysis generated ~237,000 potential neoantigens, each with a predicted MHC binding affinity across all available HLA alleles, resulting in ~20e^6^ binding affinities. From these data we aggregated all neoantigens arising from each individual mutation and filtered for neoantigens classified as strong binders (≤ 0.5% rank binding for its HLA alleles), thereby identifying those neoantigens most likely to induce an immune response resulting in their immunoediting and elimination during early tumorigenesis (Figure 1, Table S2; STAR methods in Supplementary Material). We find that 211,852 of 2,214,324 (9.6%) aggregated mutant/HLA pairs derived from cancer driver proteins are predicted to be strong binders.

**Figure 2:**
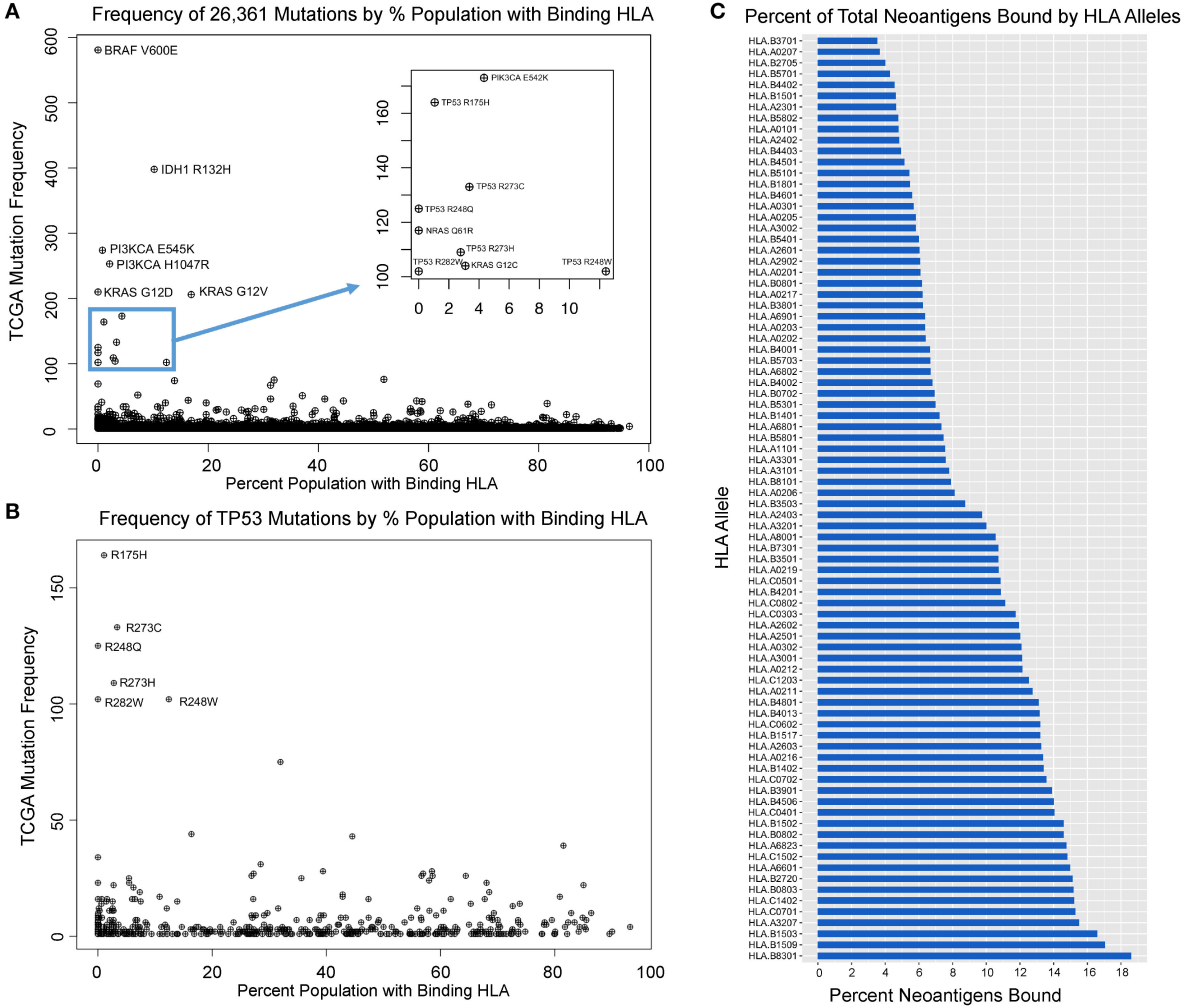
Mutation frequency in TCGA inversely correlates with population-scale HLA binding. **(A)** Mutation frequency for 26,361 TCGA variants compared to the proportion of the population predicted to bind a given neoantigens derived from that mutation reveals immunogenically silent mutations in 1,806 variants (6.85% of mutation in the TCGA, including BRAF V600E and KRAS G12D highlighted) and enrichment of common driver mutations in those which are predicted to not bind any HLA alleles (*p* = 0.0004). **(B)** TP53 mutations are enriched in those that are immunogenically silent or bind HLA alleles in a small portion of the population, and mutations with high probability of binding at least one allele in a given patient are significantly underrepresented in the TCGA (*p* = 0.008). **(C)** Proportion of characterized neoantigens bound by individual HLA alleles reveals broad range in diversity of binding from 3.5% of neoantigens arising from driver genes bound by HLA-B*37:01 to 18.6% bound by HLA-B*83:01.

Upon analyzing HLA binding by neoantigens derived from each mutant variant, we observed that while the majority of mutants produced binders across multiple HLA alleles, a subset of mutations was predicted to not produce any strong binders across all of the 84 HLA alleles studied, here defined as immunogenically silent mutations. A total of 1,806 putative neoepitopes (6.85% of characterized variants) were predicted not to bind any of the 84 HLA alleles with high affinity (Figure 2A, Table S2), including the common mutations BRAF V600E and KRAS G12D, extending the observations of Marty et al. (18). For each variant, we calculated the probability of a TCGA patient carrying at least one allele capable of binding that variant (median neoantigen presented across 26.5% of the population), and found that the most common variants in the TCGA were enriched in those mutations predicted to bind HLA less frequently across the population (Figure 2A, Table S2, *p* = 0.00035), suggesting that mutations generating neoantigens capable of being presented with high affinity by multiple, or more common HLA alleles, have a higher likelihood of being eliminated through immunoediting, and thus are underrepresented in the TCGA. The highest ranking of these, MAP3K1 D3727Y, is predicted to bind MHC with high affinity in 94.5% of the population, producing various epitopes with strong binding affinity to 50 of 84 HLA alleles tested, and this mutation is only observed once in the entire TCGA dataset. Analysis across all variants deriving the most commonly mutated gene, *TP53*, reveals that common variants are highly enriched in loci that are not capable of generating neoantigens (Figure 2B, *p* = 0.008). Furthermore, our analysis reveals a wide range in the breadth of tumor neoantigens restricted to specific HLA alleles, ranging from 3.5% of neoantigens arising from driver genes bound by HLA-B^*^37:01 to 18.6% of neoantigens bound by HLA-B^*^83:01 (Figure 2C).

**Figure 3:**
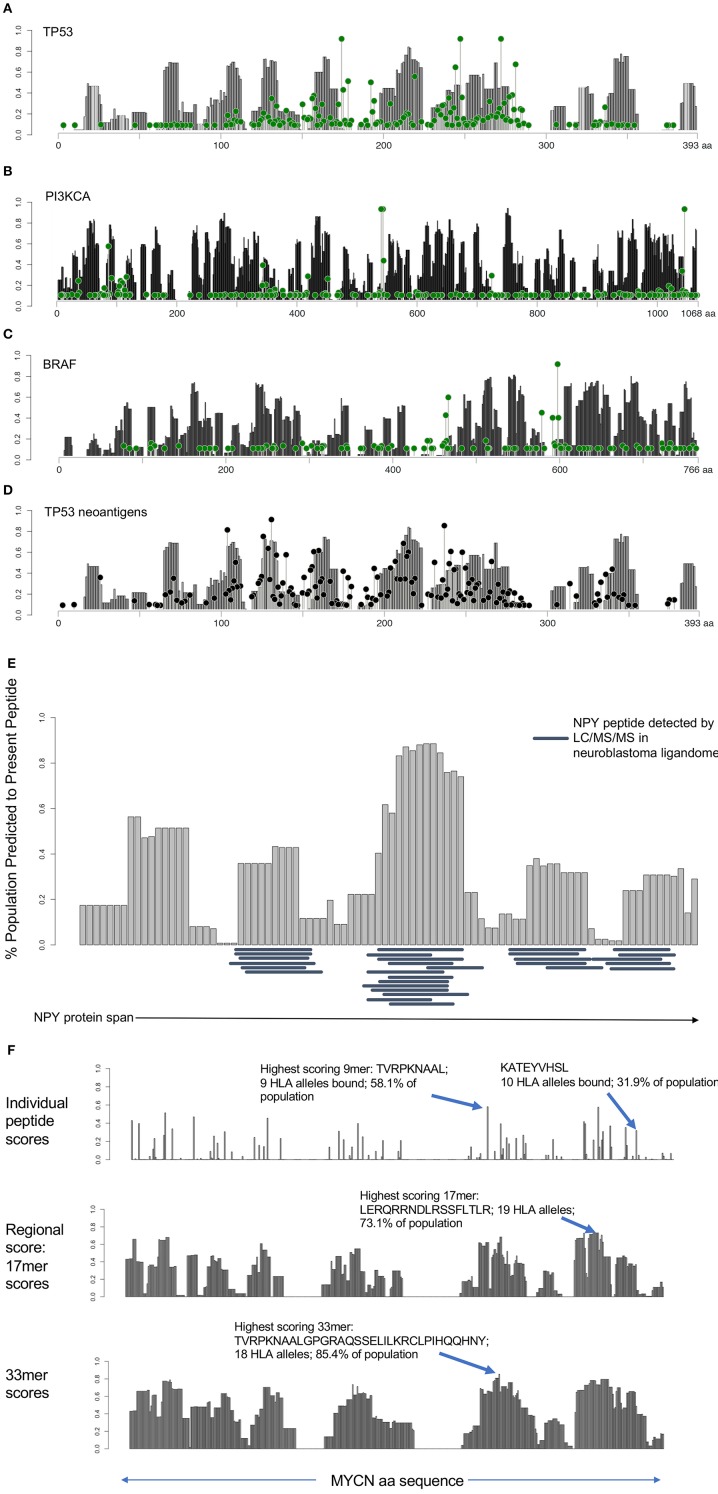
HLA presentation score by protein region reveals unprotected domains and protein regions broadly applicable as cancer vaccine candidates. **(A–C)** Regional HLA presentation scores determined by calculating the fraction of the population capable of presenting a 9 mer epitope centered at each amino acid location in p53, PI3K, and BRAF (gray bars). Each protein contains domains that are presented on HLA in the majority of the population and other domains that are unprotected. Mutation frequency at each location overlaid (green lollipop plot) reveals that many common mutations are found in unprotected regions of the protein. **(D)** Overlay of mutated neoantigen presentation score with regional presentation score for p53 shows strong correlation between regional protection of WT protein (gray bars) with protection against mutated neoantigens (black lollipop plot). **(E)** Twenty nine peptides detected by ligandomics in 16 neuroblastoma tumors were mapped onto the HLA population presentation scores. Empirically detected peptides were highly enriched in high-scoring regions of the protein (*p* = 0.000011). **(F)** Analysis of MYCN HLA presentation across the span of the protein. Analysis of individual peptides (top) reveals the most highly presented peptide derived from MYCN, TVRPKNAAL, to be presented on 9 HLA alleles, representing 58.1% of the population. KATEYVHSL peptide, detected by ligandomics is predicted to be presented on 10 total HLA alleles (31.9% of the population). Analysis of 17 mer regions (middle) reveals a peptide LERQRRNDLRSSFLTLR generating peptides predicted to bind 19 HLA alleles (73.1% of the population). Analysis of 33 mer regions reveals the highest scoring peptide TVRPKNAALGPGRAQSSELILKRCLPIHQQHNY presented on 18 HLA alleles in 85.4% of the population, suggesting these as promising regions of the MYCN protein for broadly applicable vaccination.

Having identified immunogenically silent neoepitopes, we hypothesized that HLA alleles evolved to preferentially present particular protein motifs, which may leave other regions of cancer proteins unprotected. We applied the algorithm used to generate the immunogenicity map, calculating the presentation scores of 9 mers starting at each amino acid along the span of the entire protein across 84 HLA alleles and calculating the percentage of the population predicted to present a given peptide. We then determined the combined population-wide presentation scores of the neighboring eight amino acids in either direction to calculate the regional immunity of the protein to represent all 9 mers centered at each amino acid position. We generated immune presentation maps of TP53, PI3KCA, and BRAF, and mapped this onto the frequency of point mutations at each amino acid (Figures 3A–C). We find that common hot-spot mutations frequently occur in protein domains that score low in population-scale HLA presentation, while domains that are widely protected rarely harbor recurring mutations, suggesting that mutations in unprotected regions are enriched in cancer due in part to consequent immune evasion. We also mapped the presentation score of neoantigens in TP53 onto the regional HLA presentation score (Figure 3D), observing a strong overlap between presentation scores for individual neoantigens and the corresponding wild-type protein scores, suggesting that regional scores are a good predictor of the immunogenicity of neoantigens, particularly group 1 neoantigens that do not alter the HLA restriction of the peptide.

In addition to understanding the immune evasion of proteins arising from unprotected domains of proteins, we sought to apply regional HLA presentation to identifying shared tumor epitopes derived from clinically-relevant oncogenes that can be broadly therapeutically applicable across the widest population of patients. We performed mass spectrometry on 16 neruoblastoma tumors to characterize the ligandome and test the predictive ability of the HLA regional scoring across the span of a protein. We mapped the regional presentation score of the most highly represented protein in the neuroblastoma ligandome, NPY (29 MHC Class I peptides detected in 16 neuroblastomas), finding a highly significant concordance between the empirically detected peptides and those regions of the protein expected to be highly presented (Figure 3E; *p* = 0.000011), and find no peptides in the ligandome derived from the signal peptide region (aa 1–28) which is cleaved from the full-length pro-NPY protein. Based on the high degree of presentation across the NPY protein across 68/84 HLA alleles, its high level of differential expression (Figure S6), and its role in promoting tumor growth (19), we prostulate that NPY is a promising candidate for vaccination strategies. Surprisingly, we find that despite the elevated population presentation score in the highly presented regions, none of the peptides presented in these regions are predicted to bind to HLA-A^*^02:01, highlighting the utility of a population-scale analysis of HLA presentation in identifying broadly presented epitopes that may be overlooked due to lack of presentation by the most common HLA alleles. We next searched the neuroblastoma immunopeptidomics dataset we created for peptides derived from the *MYCN* oncogene, a major cancer driver in neuroblastoma, finding only a single peptide (KATEYVHSL) presented on the relatively rare HLA-C^*^16:01 allele representing <5% of the population (Figure 3F). Applying the HLA protein scoring map, we find that this peptide is predicted to bind strongly to 10/84 HLA alleles, representing 31.9% of the population (ranking 15th of 456 peptides in population binding score), and suggesting that this peptide can have significantly broader application as a therapeutic target in this pediatric cancer population. This peptide overlaps with the previously reported immunogenic HLA-A^*^02:01 peptide VILKKATEYV (20), suggesting that immunization using this region of MYCN may have wider implications beyond HLA-A^*^02:01 patients. Using our analysis, we find that the highest scoring MYCN peptide (TVRPKNAAL) has predicted binding to 9 HLA alleles, representing 58.1% of the population, and we expect analysis of more neuroblastoma tumor specimens will validate this prediction. We further analyzed regional scores across 17 and 33 mers, we find that these regions are predicted to generate peptides binding to 73.1 and 85.4% of the population, respectively (Figure 3F). We suggest that these tools can be used to design and prioritize more broadly applicable therapeutic targets and vaccines for cancer, particularly when paired with ligandomics data (21). Analyses of population-scale presentation along the span of individual proteins and of specific neoantigens is available through the Shiny-NAP web application (http://reslnmaris01.research.chop.edu:3838/shinyNAP/).

### HLA Allelic Immunogenicity and Cancer Susceptibility

**Figure 4:**
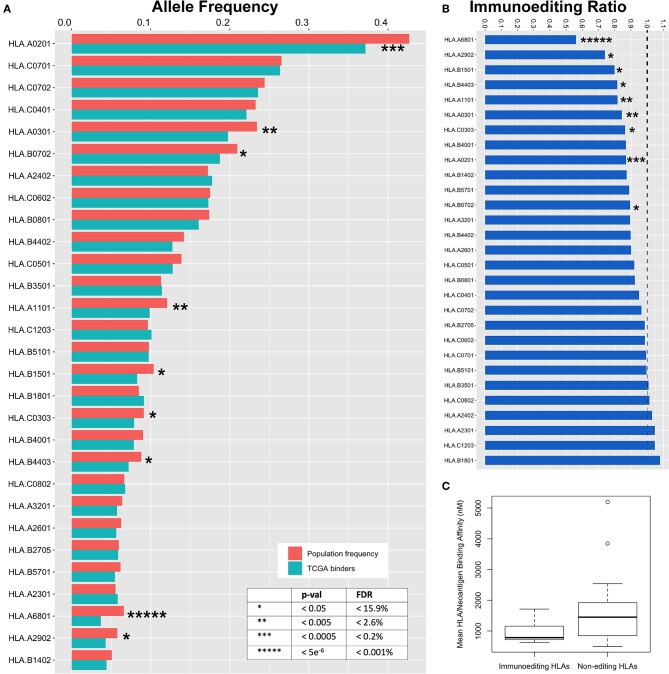
Immunoediting across HLA alleles. **(A)** Expected TCGA frequency for each allele as calculated from the Bone Marrow Registry and adjusted for ethnicity (red) compared to observed HLA allele frequency in subset of the TCGA population harboring strong neoantigen/HLA binding pairs (blue). To calculate observed HLA frequencies, predicted mutant binders are used to filter TCGA data and HLA frequencies are deduced from the subset of unique individuals. Decreased frequency in TCGA binders compared to expected frequency is a surrogate metric for early immunoediting events that are responsible for having cleared early tumors and are thus underrepresented in TCGA data. **(B)** Ratio of observed:predicted binders. Area below dotted line represents immunoediting frequency for each allele. Zero represents complete immunoediting while 1 represents no contribution of immunoediting by a particular HLA allele. **(C)** Binding affinity between HLA alleles found to participate in immunoediting of cancer neoantigens is stronger than non-contributing alleles (*p* = 0.015).

We hypothesized that specific HLA alleles capable of generating strongly bound neoantigens would be underrepresented in the population of TCGA patients harboring those variants due to early immune based elimination of neoplastic cells. To validate this prediction, we inferred HLA haplotypes from individual patient sequencing data using the PHLAT HLA typing algorithm (22), resulting in 563 unique HLA alleles characterized across the TCGA. To assure predication accuracy, we directly genotyped 15 unique HLA allele calls with the lowest confidence predictions using DNA from cancer cell lines and showed 100% concordance (Table S3). We then used the immunogenicity map to determine neoantigen binding across all TCGA driver mutations as defined above (Table S4). To estimate the degree of immunoediting across each HLA allele, we used the immunogenicity map to generate a list of all predicted strong binders at each HLA allele having greater than 5% population frequency in the TCGA (29/84 alleles), subset the unique set of patients harboring these mutations, and compared the frequency of their HLA alleles to predicted in the TCGA population from the Bone Marrow Registry data derived largely from younger adults (Figure S2). Performing this analysis across 29 HLA alleles, we calculated the magnitude of immunoediting for each allele by the underrepresentation of that allele in the subset of patients harboring strong neoantigens for that allele, as compared with the predicted TCGA population frequency calculated from the bone marrow registry data (Figure 4A). As a metric of immunoediting by each allele, we calculated the proportion of observed HLA frequencies in the neoantigen-harboring cases compared to the population frequency, in which a proportion of 0 would represent perfect immunoediting of all early neoantigens and 1 would represent no immunoediting (Figure 4B; STAR methods in Supplementary Material).

We observe a wide range of immunoediting across the 29 HLA alleles, the highest of which was HLA-A^*^68:01 with a 44% underrepresentation in the subset of patients harboring predicted binders. We also found that many common HLA alleles appear to not contribute significantly to immunoediting in cancer. Nine of 29 HLA alleles were significantly associated with a protective effect against early neoantigens (Figure 4, Table S2; *p* = 7.7e^−6^-0.05, FDR = 0.0001–15.9%). Our modeling suggests that individual HLA alleles have differential ability to initiate an immune response capable of clearing early oncogenes, and across differential breadths of variants. We generated an immunoediting score to determine the degree to which each HLA allele was protective, factoring both breadth and magnitude (% neoantigens bound ^*^ % allele underrepresentation), and determined HLA-A^*^68:01 to score the highest in protectivity against mutations in cancer driver genes (Figure S3A, Table S2). We postulated that the binding affinity between those HLA alleles and tumor neoantigens may contribute to immunoediting. By comparing the nine HLA alleles that contribute to immunoediting to the 21 alleles that were found to not contribute significantly to immunoediting, we find that the mean binding among neoantigens in the alleles contributing to immunoediting is 982.8 nM, significantly stronger than 1,612.6 nM in non-contributing alleles (Figure 4C, *p* = 0.015), suggesting that differential affinity for peptides among HLA may contribute to their ability to initiate an anti-tumor response against neoantigens. Furthermore, we scored the contribution of these alleles at a population level as a metric for how many neoantigens these alleles are editing at a population scale by calculating the product of the immunoediting score with the frequency of the HLA allele in the US population (Figure S3B, Table S2), with HLA-A^*^02:01 emerging as the most significant contributor to population-scale immunoediting.

### Immunoediting in TCGA Across Patients, Tumors, and Point Mutations

We next focused on immunogenically silent mutations defined above and showed that these are enriched in the TCGA dataset, comprising 9.1% of observed mutations, while only representing 6.85% of all characterized variants (*p* = 2e^−16^). Indeed, we find that 1,974 of 7,300 patients (27%) in the TCGA harbor at least one immunogenically silent mutation. In addition to immunoediting contributions of HLA alleles and evasion through immunogenically silent mutations, we explored editing events across individual patients, tumor types, and specific point mutations. To model the degree of immunoediting across these variables, we calculated the expected frequency with which a given patient, tumor, or point mutation, should harbor at least one HLA allele capable of binding the subset of strongly binding neoantigens in that group, calculated the collective probabilities of the subpopulation generating neoantigens, and compared the observed frequencies to the predicted frequency of binding to at least one HLA allele. To simplify the modeling of predicted allele frequencies, we treated HLA allele frequencies as independent events and disregarded linkage between HLA alleles, thus slightly underestimating the expected frequencies and biasing our prediction of immunoediting toward a more conservative estimate. These analyses lend insight into tumor cell extrinsic mechanisms for recurrent hot spot mutations in cancer (Table S5). For example, while KRAS G12D is immunogenically silent and is predicted to bind no common HLA allele with high enough affinity to allow for recognition by T cells, the KRAS G12A mutation is predicted to generate strong neoantigens with four HLA alleles (HLA-A^*^02:05, HLA-A^*^02:06, HLA-A^*^69:01, and HLA-C^*^03:03). KRAS G12A scored as the 2nd most significantly underrepresented driver SNV of the 26,361 mutations analyzed in the TCGA and was not observed in any patient tumor sample with any of the four predicted HLA binders in its 35 occurrences (4.3 predicted neoantigen/HLA pairs, Table S5, *p* = 0.01).

We also examined immunoediting contributions of group 1 and group 2 neoantigens (as defined above) in HLA-A^*^02:01 by comparing neoantigens arising from mutations in the anchor positions at residues two and nine to those mutations outside of these residues. We found no significant difference in underrepresentation between group 1 and 2 neoantigens (Figure S4), suggesting that both types of neoantigens participate in immunoediting in the context of HLA-A^*^02:01. Analyzing immunoediting across individual patients, we found a subset of patients with disproportionate degrees of immunoediting. The most significant of these, uterine cancer patient TCGA-E6-A1LX, harbored 3.4-fold fewer immunogenic neoantigens than predicted (*p* = 3.1e^−10^). Interestingly, this patient also harbored 12 immunogenically silent mutations (ranking 3rd highest in immunogenically silent mutations out of 7300 TCGA patients, Table S6), suggesting that this patient's tumor had been subjected to significant immunoediting of secondary mutations, yet was being driven largely by immunogenically silent mutations. Although the combined cases of uterine cancer taken together are the least significantly immunoedited histology, a particular subset of these patients are highly enriched among individuals with the highest degree of immunoediting (five out of the top 10 immunoedited individuals in the TCGA), highlighting these as interesting case studies for the mechanisms underlying enhanced early immunoediting and eventual immune escape. In our analysis across histologies, we find that glioblastoma is the most significantly immunoedited in early tumor formation (Figure S5; *p* = 0.008), in line with recent evidence that these tumors do not arise in immune privileged sites (23, 24). Overall, these data illustrate differential degrees of immunoediting and immune evasion across TCGA patients, histologies, and variants, suggesting that an enrichment in immunogenically silent mutations may be driving the evolution of tumors in otherwise immunocompetent individuals.

## Discussion

Here we describe a model for quantifying immunoediting during early tumorigenesis that provides insight into immunologic contributions to recurrent somatic mutation hotspots observed in human cancer, as well as immunologic contributions to cancer susceptibility. The model described herein employs orthogonal methods to recent studies in demonstrating evidence of immunoediting in the TCGA cohort (18, 25). Using an HLA-based hypothesis, we converge on the conclusion that common driver mutations evade the immune system and provide a population-scale HLA-centric basis for their overrepresentation in human cancer. In each of these studies, the immunoediting process is demonstrated to be imperfect, which can in part be explained by the false-positive predicted peptides included in the analysis which dilute the contribution of peptides actually presented, but also raises questions about disparities in immune responses. Here we provide methods that can be employed to elucidate immunoediting across HLA alleles, patients, individual variants, and other genomic or clinical features. We think that our HLA-centric population-scale model provides a baseline of comparison against which we can estimate the degree of immunoediting, with several examples of disparities across these features highlighted in this manuscript.

This is the first report that we are aware of to map known driver neoantigens across common HLA alleles, and quite strikingly the most recurrent hotspot mutations in human cancer are predicted to bind no common HLA allele with high enough affinity to engage the adaptive immune system, highlighting an immunologic mechanism underlying the evolutionary advantage of common mutations in addition to their oncogenic potency. This is also the first report of which we are aware of to quantify the contributions of individual HLA alleles to the immunoediting process in cancer, revealing a high disparity in immune protection against cancer across the HLA alleles. Our data suggest that the ability of individual HLA alleles to bind cancer neoantigens with high affinity is strongly associated with its ability to contribute to cancer immunoediting. Though our data support the cancer immunoediting theory, we and others show that the immunoediting process leads to incomplete elimination of neoantigens arising from early driver mutations. Our findings suggest that a significant difference in p/MHC binding affinities contributes to the disparity in immunoediting among HLA alleles, but further studies will be necessary to corroborate these findings and uncover additional mechanisms contributing to these disparities. While we demonstrate that a significant degree of immune evasion may be attributed to immunogenically silent mutations, the absence of complete immunoediting in patients not harboring immunogenically silent mutations may be attributed to factors including tumor intrinsic immune evasion, downregulation of MHC, lack of T cell response of the proper magnitude and quality, poor TCR repertoire, exclusion of T cells from tissue, or peripheral tolerance. We believe that our model can be coupled with genomic surrogates of these features to interrogate these variables in future studies using tumor genomic data.

Here we show that not all HLA alleles are found to be significantly protective against the neoantigens that they present. We postulate that alleles not found to significantly participate in immunoediting may induce sublethal T cell responses or possess biophysical and/or geometric properties that confer suboptimal interactions with germline-encoded binding regions of the TCR. Our findings that specific regions of cancer driver proteins are unprotected by HLA presentation combined with the disparity in binding across HLA alleles raises the question of whether HLA alleles have evolved to confer protectivity against particular viral domains that coincide with motifs found in cancer proteins, and whether the unpresented areas remain unprotected due to lack of evolutionary pressure on these motifs. These results also raise the question of whether HLA presentation of group 2 neoantigens is associated with mutational signatures arising from particular groups of DNA damage that generate variants with more favorable binding properties in the anchor residues (26). We have made available the tools for other investigators to test these and other hypotheses (http://reslnmaris01.research.chop.edu:3838/shinyNAP/).

We also present a tool for mapping the presentation scores across the span of any given protein in the population. We find a highly significant concordance between the peptides empirically detected in the combined ligandome of 16 neuroblastoma tumors carrying various HLA alleles, and the regions of the NPY protein predicted to be most highly presented by HLA across the population. Based on these results paired with the high level of differential expression, and its role in promoting tumor growth, we suggest that NPY is a promising candidate for vaccine development for neuroblastoma patients. Using this tool, we also suggest that current vaccination strategies used against MYCN may have broader application across the population. As efforts are being made to develop HLA-agnostic CAR T cells, specific to only the peptide and not the MHC, we believe these tools will be useful in identifying broader segments of the cancer population likely to benefit from these immunotherapies.

As access to genomic data from cancer patients continues to expand, and the peptide/MHC binding and T cell epitope prediction tools improve, this model will benefit from additional statistical power in stratifying subsets of the patient population based on molecular features occurring in smaller subpopulations. Despite the fact that our model predicted no HLA alleles binding to neoantigens derived from the KRAS G12D mutation, it was recently reported by Tran et al. (27) that KRAS G12D neoantigen GADGVGKSA is able to mediate a T cell response in the context of HLA-C^*^08:02. This antigen is predicted to be a weak HLA binder (15,390 nM), highlighting the fact that T cell epitopes are not always predicted using this algorithm, particularly on rare alleles for which there are limited training data, and that new methods will help identify neoantigens with non-canonical motifs (28). Here, we restricted our analysis of immunoediting by CD8 T cells through MHC class I presentation of 9 mer antigens only, and did not account for immunoediting that may be triggered by other Class I antigens derived from additional structural variants or non-canonical antigens, peptides of varying lengths, Class II antigens, or the activities of the innate immune system from NK cells or macrophages, as we were focused on maintaining statistical power by using common HLA alleles and the most common Class I peptides for which we were most confident in being contributors to early tumorigenesis.

We find that the highest statistically significant immunoediting takes place in glioblastoma, whereas, taken together, sarcomas, pancreatic tumors, ovarian, adrenocortical tumors and lymphomas show no significant evidence of immunoediting. Given that the immunogenically silent KRAS G12D mutation is pathognomonic of pancreatic cancer, our findings may help explain the lack of efficacy of treatments such as checkpoint inhibitors in pancreatic cancer (29) and the lack of immunoediting observed in our analysis, as these tumors are driven largely by an immunogenically silent mutation. We suggest that our methods could ultimately be used to inform the stratification of groups of patients most likely to respond to immunotherapies such as checkpoint inhibitors based on patient HLA and antigen immunogenicity, and to prioritize shared antigens for vaccine development or HLA-agnostic CAR strategies. This model can also be used to predict how an individual's HLA profile can determine the types of mutations most likely to develop or be protected against.

Using the model of immunogenicity described herein, it may be possible to infer physical properties of neoantigens that elicit high immunoediting as compared to other neoantigens that are presented but not eliminated by the immune system, study the contributions of various molecular pathways across tumor types and across individual patients that contribute to variable degrees of immunoediting, and as a basis for exploring the mechanisms by which specific HLA alleles may contribute to cancer protection and predisposition. With alleles such as HLA-A^*^68:01 emerging as disproportionately high in their immunoediting score, we will be interested to see whether contributions of HLA alleles to early immunoediting will translate to improved abilities to induce T cell responses against tumor neoantigens in patients, and whether such alleles are associated with improved outcomes in patients treated with modern immunotherapies. We suggest that the immunogenicity map, HLA typing data, and immunoediting model contained herein will facilitate investigation into neoantigen immunogenicity at the level of HLA alleles, mutations, patients, histologies, and aid in prioritization of shared tumor epitopes for therapeutic development, and further our mechanistic understanding of immune evasion in tumor evolution.

## Data Availability Statement

The datasets generated for this study can be found in the TCGA and Analyses of population-scale presentation along the span of individual proteins and of specific neoantigens is available through the Shiny-NAP web application (http://reslnmaris01.research.chop.edu:3838/shinyNAP/).

## Author Contributions

MY and JM: conceptualization, writing, and supervision. MY, PR, MM, SS, DM, HL, and JM: methodology. MY, AF, AS, PR, and JP: software. MY, JP and HL: formal analysis. MY and JP: investigation. MY and JM: funding acquisition.

### Conflict of Interest

The authors currently have a patent pending for methods of prioritizing tumor antigens, which is based on some of the work presented in this manuscript.

## Supplementary Material

Table S1 Driver oncogenes and tumor suppressor genes. List of 125 cancer driver genes implicated in carcinogenesis, including oncogenes and tumor suppressor genes that regulate cell fate, cell survival, and genome maintenance (16).

Table S2 Immunogenicity map of TCGA. Map of all characterized variants derived from 125 cancer driver genes (Table S1) and their binding score across each of 84 characterized HLA alleles. Binding score reported as the strongest rank score from the aggregated pool of all peptides resulting from each variant to particular HLA allele, with lower scores representing higher binding affinity relative to binders of each HLA allele.

Table S3 Validation of HLA genotyping. Comparison of HLA predictions across 15 HLA alleles as inferred from PHLAT algorithm from exome sequencing data of three neuroblastoma cell lines compared to clinical genotyping performed using next generation sequencing of amplified HLA loci.

Table S4 TCGA mutations and patient HLA types. List of variants used to analyze immunoediting across patients, variants, and histologies. Table populated with all mutations in driver genes from the TCGA with matched HLA typing inferred from PHLAT.

Table S5 Immunoedited variants from TCGA. List of variants most underrepresented when measured with population of patients harboring HLA alleles predicted to bind neoantigens derived from variant (*p* ≤ 0.05). Frequency of mutation is number of occurrences of mutation in Table S4. Percent of population with binders is the probability of a TCGA subject harboring an HLA allele capable of binding a neoepitope derived from the particular variant. Observed mutation is frequency calculated from the number of patients with at least one HLA allele from the set of those capable of binding the variant.

Table S6 Immunoedited subjects from TCGA. List of subjects with highest degrees of immunoediting in the TCGA (*p* ≤ 0.05). Expected binders calculated by summing the probability of all individual variants in each patient being bound to an HLA allele in the TCGA. Observed binders is the summed number of variant/HLA pairs that generate at least one epitope across each variant. Observed/expected represents the degree of underrepresentation of presented neoantigens in each patient (0 being perfect immunoediting). Despite being ranked the lowest in significance for immunoediting, uterine cancer represents 5 of the top 10 patients with the most significant degrees of immunoediting. The most significantly immunoedited subject also ranks 3 of 7,300 in number of immunogenically silent mutations.

Figure S1 Pipeline for inferring HLA type from TCGA and comparing to predicted frequencies. BAM files for individual patients were converted to FASTQ and processed using PHLAT to determine HLA type. HLA frequencies in TCGA were determined using ethnicity-specific allele populations from Bone Marrow Registry and compared to observed frequencies in TCGA. Patient HLA and mutation data were combined to determine number of neoantigens in each individual, allowing the comparison of predicted HLA frequencies to ethnicity-adjusted HLA frequencies in the TCGA across individuals, mutations, and tumor histologies.

Figure S2 Workflow for modeling immunoediting for individual HLA alleles (example show for HLA-A^*^02:01). All strong neoantigens predicted to bind given HLA are aggregated and used to filter the TCGA dataset. Resulting mutations are filtered for unique patients to remove patients harboring multiple binders to a single allele. Frequency of unique patients harboring at least one strong neoantigen binding to predicted HLA allele compared to ethnicity-adjusted predicted value for TCGA frequency to determine level of immunoediting by specific HLA allele.

Figure S3 HLA allele immunoediting scores and population editing scores. Immunoediting scores represent overall ability of HLA alleles to edit mutations, accounting for the repertoire of antigens they are able to bind and the level of editing that they exhibit for that subset of antigens (calculated by % neoantigens bound by allele ^*^ % underrepresentation of HLA allele), with HLA-A^*^68:01 scoring highest in immunoediting of neoantigens arising from mutations in early driver genes. Immunoediting population score is used to estimate the total immunoediting contribution of HLA alleles across the US population (calculated by the product of the immunoediting score with the US HLA allele frequency). **(A)** Immunoediting scores in HLA alleles shown to be statistically significant. **(B)** Population immunoediting scores in HLA alleles shown to be statistically significant.

Figure S4 Immunoediting of group 1 and group 2 neoantigens. Neoantigens resulting from group 1 neoantigens (those with neoantigens occurring from mutation at positions outside of anchor residues) were compared with group 2 neoantigens (mutations occurring at anchor residues 2 and 9) in HLA-A^*^02:01. No significant difference in underrepresentation was found between groups 1 and 2 in HLA-A^*^02:01.

Figure S5 Immunoediting by cancer histology. Combined observed binding neoantigens compared to expected. Zero represents complete immunoediting while one represents no contribution of immunoediting by a particular HLA allele. Glioblastoma is the only significantly immunoedited histology in this analysis (*p* = 0.008). Uterine cancer is the least significantly immunoedited tumor, though is highly enriched in individuals exhibiting high degrees of immunoediting (4/8 of the most significantly edited patients in the TCGA, Table S6).

Figure S6 NPY is highly differentially expressed in neuroblastoma and is a promising target for vaccination. RNA-sequencing data from 153 neuroblastoma tumors in TARGET (first column) compared to 1,643 normal tissues from GTEx compiled by organ (subsequent columns) reveals high expression of NPY in neuroblastoma as compared to normal tissues.
