# Supplementary material for: Immunogenicity and Immune Silence in Human Cancer
Source: Front Immunol. 2020 Mar 6;11:69. doi: 10.3389/fimmu.2020.00069 (PMC7092187; doi:10.3389/fimmu.2020.00069)
Supplement: Supplementary file 1 [file Data_Sheet_1.docx]

**STAR Methods**

**CONTACT FOR REAGENT AND RESOURCE SHARING**

Further information and requests for resources and reagents should be directed to and will be fulfilled by the Lead Contact, John Maris (maris@chop.edu).

**EXPERIMENTAL MODEL AND SUBJECT DETAILS**

**TCGA subjects**

7300 TCGA subjects were used in analysis based on cases sequences using Illumia HiSeq 2000. HLA-A*01:01 was excluded from analysis due to ambiguous calls made by PHLAT algorithm for this allele, and HLA-E alleles were excluded.

**METHODS DETAILS**

**HLA typing**

BAM files from TCGA were converted to FASTQ using Bedtools and subsequently processed using PHLAT algorithm. Pipeline was created on Cavatica and processing was performed on Seven Bridges Genomics cloud server. Subsequent analysis was performed using R Studio.

**Immunogenicity map of all driver mutation in TCGA**

To generate a map of immunogenicity across all initiating cancer events, TCGA mutants arising from 125 early tumor driver genes imputed by MuSE and Somatic Sniper were used to generated amino acid sequences for all possible 9mer peptides resulting from mutant amino acid plus flanking sequences. Binding affinities were predicted using NetMHC-4.0 across 87 common HLA alleles. HLA alleles not occurring in the TCGA and HLA-E alleles were removed from the analysis, resulting in 84 HLA alleles. Combined binding affinities across all resulting peptides (ie. those resulting from multiple of 9 potential epitopes) for each variant were aggregated, then filtered for antigens producing epitopes most likely to be recognized by T cells (≤0.5% allele rank).

**HLA immunoediting model**

To model early immunoediting, we compared the frequency of HLA alleles in the population of patients harboring mutations expected to yield a strong neoantigen to the HLA allele frequency in the Bone Marrow Registry (largely derived from young adults) after adjusting for ethnic distribution within the TCGA population. To estimate the degree of immunoediting across each HLA allele, we used the immunogenicity map to generate a list of all predicted strong binders, subset all TCGA patients harboring at least one of the mutations, filtered for unique patients harboring these mutations, and compared the frequency of their HLA alleles to that of predicted TCGA frequency. TCGA population frequency was calculated by matching ethnicity-specific HLA allele frequencies from the Bone Marrow Registry to the representation of these ethnicities in the TCGA.

**Prediction of expected HLA binders**

To calculate predicted HLA binders for specific mutant variants, tumor types, and individual patients, we calculated the probability of an individual not possessing at least one binding allele, and subtracted this probability from 1 using the formula, which gives the probability that an individual carrying at least one such a binding allele,

1 – $\prod_{k=1}^{K} (1-p\_k)$

where k $\in$ set of K binding alleles and *p_k* is the probability of individual carrying at least one copy of the allele k. Note that this probability calculation assumes that these K binding alleles occur independently in the population. Dependency among these K binding alleles due to positive linkage disequilibrium (Maiers et al., 2007) may lead to over-estimate of the true probability, which may make our estimate of immunoediting conservative.

Expected occurrence of HLA allele in population of mutants, patients, and tumors was calculated using the formula:

$$\sum_{j=1}^{J} (1-\prod_{k=1}^{K\_j} (1-P\_k))$$

where k $\in$ set of K_j binding alleles, *P_k* is the probability of individual carrying at least one copy of the allele *k,* and j $\in$ set of J mutants, patients, or tumors, respectively.

p values were calculated using one-sided binomial test to compare the observed number of binders with expected based on the population frequency using R and false discovery rate (FDR) was calculated using the Benjamini/Hochberg method to adjust for multiple comparisons.

**Isolation of HLA ligands by immunoaffinity purification**

Eight patient-derived xenograft tumors and eight primary patient tumors were lysed in 10 mM CHAPS/PBS (AppliChem /Lonza) containing 1x protease inhibitor (Complete; Roche, Basel, Switzerland). Mouse MHC molecules were reduced using a 1 h immunoaffinity purification with H-2K-specific mAb 20-8-4S, covalently linked to CNBr-activated sepharose (GE Healthcare, Little Chalfont, UK). Remaining HLA molecules were purified overnight using the pan-HLA class I-specific mAb W6/32 or a mix of the pan-HLA class II-specific mAb Tü39 and the HLA-DR-specific mAb L243, covalently linked to CNBr-activated. MHC-peptide complexes were eluted by repeated addition of 0.2% trifluoroacetic acid (Merck). Elution fractions E_1_-E_4_ were pooled and free MHC ligands were isolated by ultrafiltration using centrifugal filter units (Amicon; Merck Millipore). MHC ligands were extracted and desalted from the filtrate using ZipTip C_18_ pipette tips (Merck Millipore). Extracted peptides were eluted in 35 µl of acetonitrile (Merck)/0.1% trifluoroacetic acid, centrifuged to complete dryness and resuspended in 25 µl of 1% acetonitrile/0.05% trifluoroacetic acid. Samples were stored at ‑20°C until analysis by LC-MS/MS.

**Analysis of HLA ligands by LC-MS/MS**

Peptide samples were separated by reversed-phase liquid chromatography (nanoUHPLC, UltiMate 3000 RSLCnano, Dionex) and subsequently analyzed in an on-line coupled Orbitrap Fusion Lumos (Thermo Fisher Scientific). Samples were analyzed in 3 technical replicates. Sample volumes of 5 µl (sample shares of 20%) were injected onto a 75 µm x 2 cm trapping column (Acclaim PepMap RSLC, Dionex) at 4 µl/min for 5.75 min. Peptide separation was subsequently performed at 50°C and a flow rate of 300 nl/min on a 50 µm x 25 cm separation column (Acclaim PepMap RSLC, Dionex) applying a gradient ranging from 2.4-32.0% of acetonitrile over the course of 90 min. Eluting peptides were ionized by nanospray ionization and analyzed in the mass spectrometer implementing the *TopSpeed* method. Survey scans were generated in the Orbitrap at a resolution of 120,000. Precursor ions were isolated in the quadrupole, fragmented by either collision induced dissociation (CID) in the dual-pressure linear ion trap for MHC class I-purified peptides or higher-energy collisional dissociation (HCD) for MHC class II-purified peptides in the ion-routing multipole. Finally, fragment ions were recorded in the Orbitrap. For fragmentation mass ranges were limited to 400-650 m/z with charge states 2+ and 3+ for MHC class I or 300-1500 m/z with charge states 2+ to 5+ for MHC class II, respectively.

**Database Search and Spectral Annotation**

Data was processed against the human proteome as comprised in the Swiss-Prot database ([www.uniprot.org](http://www.uniprot.org), release: September 27th 2013; 20,279 reviewed protein sequences contained) using the SequestHT algorithm in the Proteome Discoverer (v1.3, ThermoFisher) software. [For the non-canonical peptide search data was processed against sample-specific fasta files created from RNA-Seq reads.] Precursor mass tolerance was set to 5 ppm, fragment mass tolerance to 0.02 Da. Search was not restricted to an enzymatic specificity. Oxidized methionine was allowed as a dynamic modification. False discovery rate (FDR) was determined by the Percolator algorithm based on processing against a decoy database consisting of shuffled sequences. FDR was set at a target value of q≤0.05 (5% FDR). Peptide-spectrum matches (PSMs) with q≤0.05 were filtered according to additional orthogonal parameters to ensure spectral quality and validity. Peptide lengths were limited to 8-12 amino acids for MHC class I and 8-25 aa for MHC class II. HLA annotation was performed using SYFPEITHI and NetMHC-4.0 for HLA class I or NetMHCIIpan for HLA class II, respectively.
