# Supplementary figures and images for: Immunogenicity and Immune Silence in Human Cancer
Source: Front Immunol. 2020 Mar 6;11:69. doi: 10.3389/fimmu.2020.00069 (PMC7092187; doi:10.3389/fimmu.2020.00069)

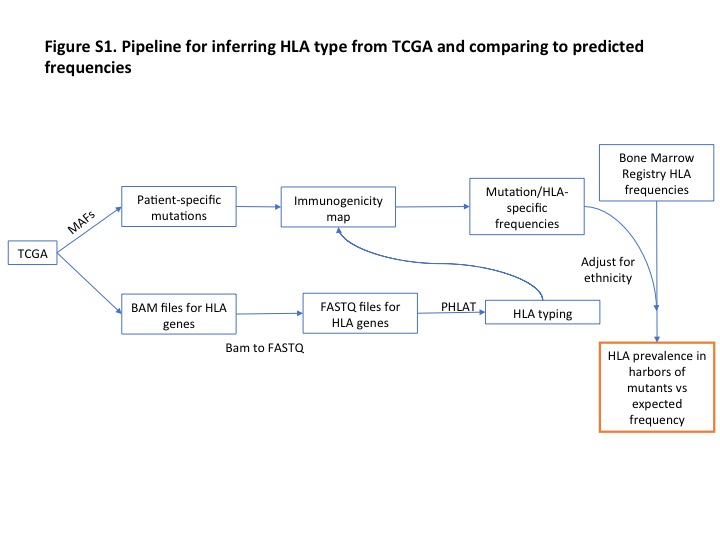

Supplement: Figure S1 — Pipeline for inferring HLA type from TCGA and comparing to predicted frequencies. BAM files for individual patients were converted to FASTQ and processed using PHLAT to determine HLA type. HLA frequencies in TCGA were determined using ethnicity-specific allele populations from Bone Marrow Registry and compared to observed frequencies in TCGA. Patient HLA and mutation data were combined to determine number of neoantigens in each individual, allowing the comparison of predicted HLA frequencies to ethnicity-adjusted HLA frequencies in the TCGA across individuals, mutations, and tumor histologies. [file Image_1.JPEG]

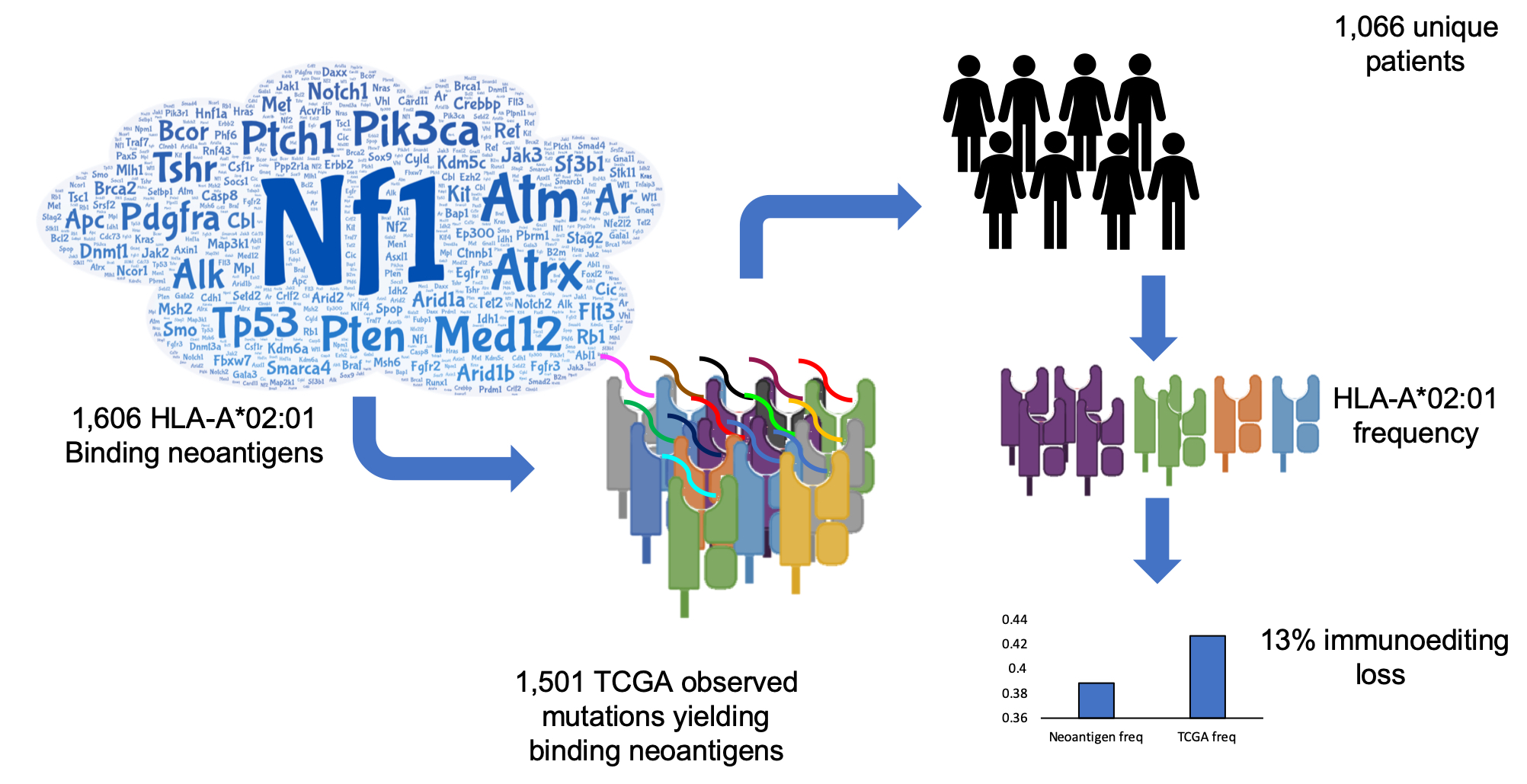

Supplement: Figure S2 — Workflow for modeling immunoediting for individual HLA alleles (example show for HLA-A*02:01). All strong neoantigens predicted to bind given HLA are aggregated and used to filter the TCGA dataset. Resulting mutations are filtered for unique patients to remove patients harboring multiple binders to a single allele. Frequency of unique patients harboring at least one strong neoantigen binding to predicted HLA allele compared to ethnicity-adjusted predicted value for TCGA frequency to determine level of immunoediting by specific HLA allele. [file Image_2.jpg]

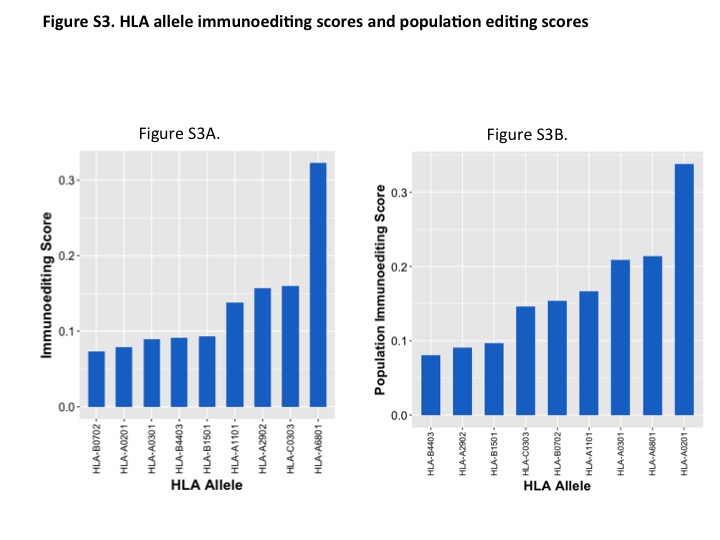

Supplement: Figure S3 — HLA allele immunoediting scores and population editing scores. Immunoediting scores represent overall ability of HLA alleles to edit mutations, accounting for the repertoire of antigens they are able to bind and the level of editing that they exhibit for that subset of antigens (calculated by % neoantigens bound by allele * % underrepresentation of HLA allele), with HLA-A*68:01 scoring highest in immunoediting of neoantigens arising from mutations in early driver genes. Immunoediting population score is used to estimate the total immunoediting contribution of HLA alleles across the US population (calculated by the product of the immunoediting score with the US HLA allele frequency). (A) Immunoediting scores in HLA alleles shown to be statistically significant. (B) Population immunoediting scores in HLA alleles shown to be statistically significant. [file Image_3.JPEG]

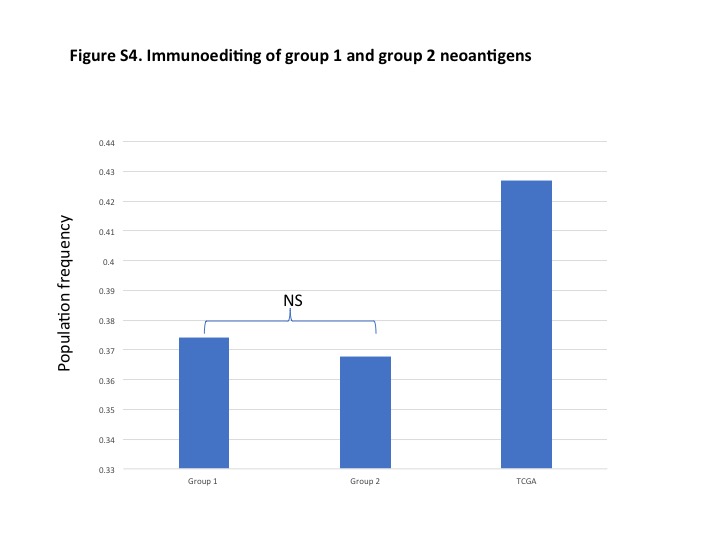

Supplement: Figure S4 — Immunoediting of group 1 and group 2 neoantigens. Neoantigens resulting from group 1 neoantigens (those with neoantigens occurring from mutation at positions outside of anchor residues) were compared with group 2 neoantigens (mutations occurring at anchor residues 2 and 9) in HLA-A*02:01. No significant difference in underrepresentation was found between groups 1 and 2 in HLA-A*02:01. [file Image_4.JPEG]

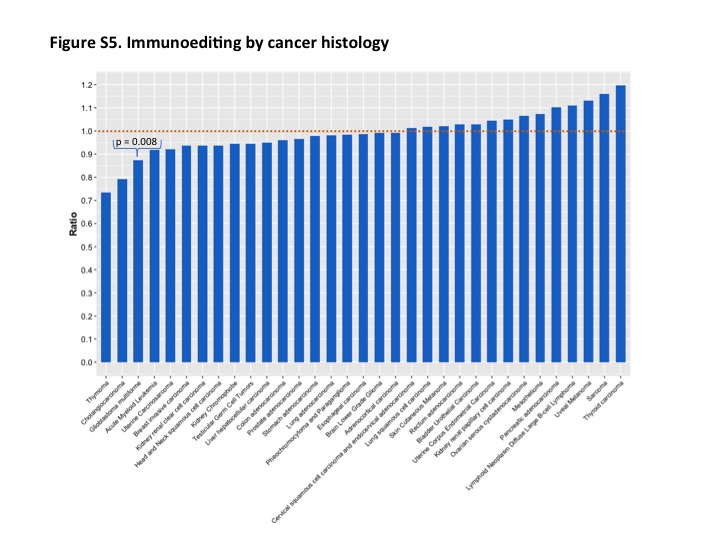

Supplement: Figure S5 — Immunoediting by cancer histology. Combined observed binding neoantigens compared to expected. Zero represents complete immunoediting while one represents no contribution of immunoediting by a particular HLA allele. Glioblastoma is the only significantly immunoedited histology in this analysis (p = 0.008). Uterine cancer is the least significantly immunoedited tumor, though is highly enriched in individuals exhibiting high degrees of immunoediting (4/8 of the most significantly edited patients in the TCGA, Table S6). [file Image_5.JPEG]

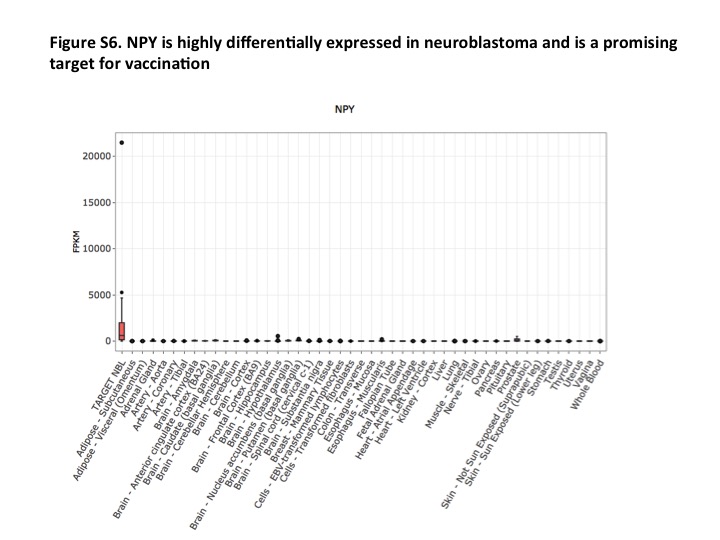

Supplement: Figure S6 — NPY is highly differentially expressed in neuroblastoma and is a promising target for vaccination. RNA-sequencing data from 153 neuroblastoma tumors in TARGET (first column) compared to 1,643 normal tissues from GTEx compiled by organ (subsequent columns) reveals high expression of NPY in neuroblastoma as compared to normal tissues. [file Image_6.JPEG]
